# Identification of malignant early repolarization pattern by late QRS activity in high‐resolution magnetocardiography

**DOI:** 10.1111/anec.12741

**Published:** 2020-01-19

**Authors:** Naotsugu Iwakami, Takeshi Aiba, Shiro Kamakura, Hiroshi Takaki, Toshiaki A. Furukawa, Tosiya Sato, Wenxu Sun, Toshiaki Shishido, Kunihiro Nishimura, Yuko Yamada‐Inoue, Satoshi Nagase, Wataru Shimizu, Satoshi Yasuda, Masaru Sugimachi, Kengo Kusano

**Affiliations:** ^1^ Department of Cardiovascular Medicine National Cerebral and Cardiovascular Center Osaka Japan; ^2^ Department of Research Promotion and Management National Cerebral and Cardiovascular Center Osaka Japan; ^3^ Department of Health Promotion and Human Behavior Kyoto University School of Public Health Kyoto Japan; ^4^ Department of Cardiovascular Dynamics Research Institute National Cerebral and Cardiovascular Center Osaka Japan; ^5^ Department of Biostatistics Kyoto University School of Public Health Kyoto Japan; ^6^ Department of Preventive Medicine and Epidemiology Informatics National Cerebral and Cardiovascular Center Osaka Japan; ^7^ Department of Cardiovascular Medicine Graduate School of Medicine Nippon Medical School Tokyo Japan

**Keywords:** early repolarization pattern, electrocardiography, magnetocardiography, sudden cardiac death, ventricular fibrillation

## Abstract

**Background:**

The early repolarization pattern (ERP) in electrocardiography (ECG) has been considered as a risk for ventricular fibrillation (VF), but effective methods for identification of malignant ERP are still required. We investigated whether high spatiotemporal resolution 64‐channel magnetocardiography (MCG) would enable distinction between benign and malignant ERPs.

**Methods:**

Among all 2,636 subjects who received MCG in our facility, we identified 116 subjects (43 ± 18 years old, 54% male) with inferior and/or lateral ERP in ECG and without structural heart disease, including 13 survivors of VF (ERP‐VF(+)) and 103 with no history of VF (ERP‐VF(−)). We measured the following MCG parameters in a time‐domain waveform of relative current magnitude: (a) QRS duration (MCG‐QRSD), (b) root‐mean‐square of the last 40 ms (MCG‐RMS40), and (c) low amplitude (<10% of maximal) signal duration (MCG‐LAS).

**Results:**

Compared to ERP‐VF(−), ERP‐VF(+) subjects presented a significantly longer MCG‐QRS (108 ± 24 vs. 91 ± 23 ms, *p* = .02) and lower MCG‐RMS40 (0.10 ± 0.08 vs. 0.25 ± 0.20, *p* = .01) but no difference in MCG‐LAS (38 ± 22 vs. 29 ± 23 ms, *p* = .17). MCG‐QRSD and MCG‐RMS40 showed significantly larger area under the ROC curve compared to J‐peak amplitude in ECG (0.72 and 0.71 vs. 0.50; *p* = .04 and 0.03). The sensitivity, specificity, and odds ratio for identifying VF(+) based on MCG‐QRSD ≥ 100 ms and MCG‐RMS40 ≤ 0.24 were 69%, 74%, and 6.33 (95% CI, 1.80–22.3), and 92%, 48%, and 10.9 (95% CI, 1.37–86.8), respectively.

**Conclusion:**

Magnetocardiography is an effective tool to distinguish malignant and benign ERPs.

## 1 INTRODUCTION

Early repolarization pattern (ERP) characterized by an end‐of‐QRS notch or slur in 12‐lead electrocardiography (ECG) is a common ECG finding, occurring in approximately 3%–24% of the general population and particularly in young male subjects or athletes. It has been associated with ventricular fibrillation (VF) for the past decade (Haissaguerre et al., 2008; Tikkanen et al., 2009; Wu, Lin, Cheng, Qiang, & Zhang, 2013). Accumulating evidence has raised the possibility of increased arrhythmic risk in patients in whom ERP is incidentally identified through routine ECG recordings. Although some ECG characteristics, including distribution, configuration, and peak amplitude of ERP, have been reported as potential tools for stratifying the risk of VF (Tikkanen et al., 2011), there is no method to identify clinically actionable risk (Arbelo & Brugada, 2015; Mahida et al., 2015). Invasive electrophysiological studies have also failed to determine the risk of VF in patients with ERP (Mahida et al., 2015). Thus, risk stratification of ERP remains rudimentary and challenging (Arbelo & Brugada, 2015; Mahida et al., 2015).

Compared with standard 12‐lead ECG, magnetocardiography (MCG) or the detection of cardiac magnetic fields has a higher spatial resolution and has different sensitivities especially to abnormal currents consisting of tangential components (Barry et al., 1977; Nousiainen, Lekkala, & Malmivuo, 1986). In fact, late fields of the QRS complexes in MCG showed higher performance in detecting lethal arrhythmic risk in patients with prior myocardial infarction than signal‐averaged ECG (SAECG; Korhonen et al., 2000, 2002). Localization of abnormal current sources has also been challenged (Leder et al., 2001). This study investigated whether high spatiotemporal resolution 64‐channel MCG could also reveal arrhythmogenicities of ERP and could be used as a means of distinguishing malignant ERPs from benign ones.

## 2 METHODS

### 2.1 Study population

The source population consisted of 2,636 consecutive subjects who had undergone MCG at the National Cerebral and Cardiovascular Center, Osaka, Japan, between April 2007 and March 2014, including subjects with or without cardiovascular disease of any kind. MCG was applied in daily clinical practice for any subjects visiting the hospital, just like ECG. From among them, we enrolled all subjects in whom the 12‐lead ECG showed inferior and/or lateral ERP as defined in a 2015 consensus paper (Macfarlane et al., 2015), and who had no structural or electrical heart disorders, according to the criteria used by Haissaguerre et al. (2008) Diagnosis of idiopathic VF was based on documented VF and exclusion of structural and electrical heart disorders by echocardiography, cardiac magnetic resonance imaging, and coronary angiography. Electrical disorders were defined as present if the patient was taking any antiarrhythmic drugs or had the following ECG findings at rest or on drug and exercise testing: QRS wider than 120 ms or bundle branch blocks, spontaneous or drug‐induced type‐1 Brugada ECG in the right precordial leads (V_1_ to V_3_) and/or higher costal leads, long or short QT syndrome with corrected QT interval (QTc) of more than 440 ms or <340 ms, and catecholaminergic polymorphic ventricular tachycardia. As for non‐VF subjects, we excluded structural and electrical disorders following the clinical diagnosis. This population was composed of healthy volunteers without any cardiac symptoms and those with complaints or symptoms but without identifiable cardiac disorders after thorough examinations. History of syncope was identified when subjects had experienced unexplained transient loss of consciousness characterized by a rapid onset, short duration, and spontaneous complete recovery. For reference, we additionally enrolled subjects without heart disorder or ERP in ECG from the source population to compare the MCG parameters.

The institutional review board at the National Cerebral and Cardiovascular Center approved this MCG study with waivers of individual consent (M23‐050, M24‐050‐6). The ethics committee at Kyoto University, where the analysis was conducted, approved the use of the cohort data for this analysis (E2321). The research was conducted in accordance with the Declaration of Helsinki.

### 2.2 12‐lead ECG and SAECG

The details of the ECG and SAECG protocols are described in the Appendix S1. In brief, we obtained the following ECG data in all patients: distribution, configuration, and peak amplitude of the ERP; ST morphology; T‐wave/R‐wave (T/R) ratio; and QRS and QTc interval as defined in a 2015 consensus paper (Macfarlane et al., 2015). ERP distribution was classified as described in a previous paper (Antzelevitch & Yan, 2010), with type 1 consisting of lateral ERPs, type 2 of inferolateral ERPs, and type 3 of inferolateral and right precordial ERPs, though type 3 was not included in this study. The T/R‐amplitude ratio was calculated in lead II and V_5_ (Roten et al., 2016).

### 2.3 MCG parameters

**Figure 1:**
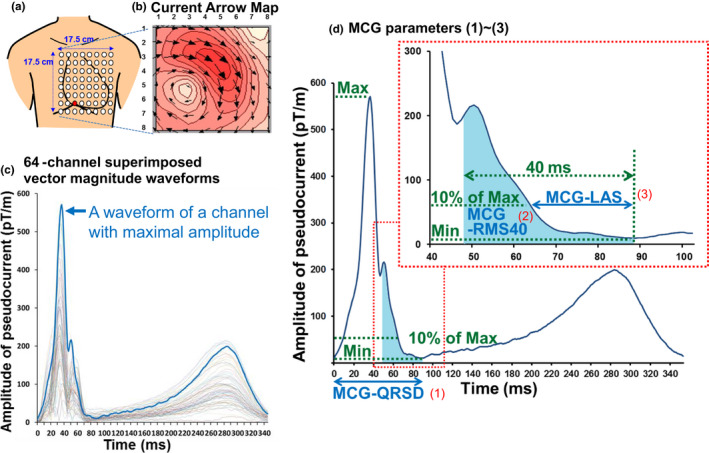
MCG study and definition of MCG parameters. (a) 64 (8 × 8) SQUID sensors arranged with 25 mm pitch were placed close to the chest in a supine position. (b) After signal filtering and baseline correction, the measured magnetic fields were mathematically transformed into pseudoelectrical currents. Presenting map corresponds to the moment of peak amplitude. (c) Time‐domain waveforms of current magnitudes in 64 channels were superimposed, and the maximal peak amplitude channel was used for analysis. (d) Definition of the three parameters at the terminal QRS complex. QRS end was defined as the minimal amplitude point. (1) MCG‐QRSD: the QRS duration. (2) MCG‐RMS40: the root‐mean‐square amplitude of the terminal 40 ms corrected by the maximal amplitude. (3) MCG‐LAS; the duration of low signal amplitude at the terminal QRS under 10% of maximal amplitude. ECG, electrocardiography; LAS, low signal amplitude; Max, maximal amplitude; MCG, magnetocardiography; Min, minimal amplitude; RMS, root‐mean‐square; SQUID, superconducting quantum interference device

Details of the method of MCG recording have been previously described (Kandori et al., 2008; Kawakami et al., 2016) and are shown in the Appendix S1 and Figure S1. In brief, we used a 64‐channel MCG system (MC‐6400, Hitachi High‐Technologies Ltd.) with highly sensitive superconducting quantum interference device (SQUID) sensors arranged in an 8 × 8 matrix with 25‐mm pitch and a 175 × 175 mm measurement area (Figure 1a). The detected signals were passed through an analog bandpass filter (0.1–100 Hz) and an analog notch filter (60 Hz). They were subsequently digitized at a sampling rate of 1 kHz by an analog–digital converter. To remove the noise in the signals, the MCG data were signal‐averaged 30 times using a trigger of simultaneously recorded ECG signals. The tangential component of measured magnetic fields (Bz) was then mathematically transformed into pseuCdoelectrical currents (C) by the Hosaka–Cohen transformation (Hosaka & Cohen, 1976; Figure 1b):

$$\vec{C}=\frac{\partial B_{z}}{\partial y}{\vec{e}}_{x}-\frac{\partial B_{z}}{\partial x}{\vec{e}}_{y}$$

where *x* and *y* (and *z*) are axes fixed in the torso with *x* directed from right to left and *y* from head to toe. The unit vectors *x* and *y* direction were described as *e_x_* and *e_y_*. Time‐domain waveforms of their magnitudes were plotted for each channel (Figure 1c). We focused on the waveform with maximal amplitude. We defined the end of the QRS complex as the minimal amplitude point, and the onset was automatically determined by the simultaneously recorded ECG (Figure 1d). We then measured the following three parameters: (a) MCG‐QRSD: the interval between the onset and end‐of‐QRS complex, (b) MCG‐RMS40: the root‐mean‐square (RMS) amplitude of the terminal 40 ms divided and corrected by the maximal amplitude, and (c) MCG‐LAS: the duration of low amplitude signal (LAS) at the terminal portion of the QRS complex under 10% of maximal amplitude. All parameters were automatically calculated from raw data by an original computing program with a dedicated code.

### 2.4 Statistical analyses

All continuous variables were shown as means (standard deviation, *SD*) or medians (interquartile range, IQR), as appropriate. We examined the association between groups and variables using unpaired *t* test for continuous variables and Fisher's exact test for categorical variables. We performed univariable and multivariable logistic regression analyses to compare ECG and MCG parameters. In multivariable analysis, MCG‐QRSD represented the conceptually interrelated three MCG parameters to avoid multicollinearity because it alone can be determined without defining thresholds of 40 ms or 10%. We used Pearson's correlation coefficients to examine the correlations among parameters. We further compared the area under the receiver operating characteristic (ROC) curve of the parameters with continuous value by DeLong test, using the representative ECG predictor of J‐peak amplitude as a reference (Rosso et al., 2008; Tikkanen et al., 2009, 2011). We also calculated measures of accuracy to identify malignant ERPs such as sensitivity, specificity, positive and negative likelihood ratios, and odds ratio. We used previously reported representative cutoff values for existing ECG parameters such as J‐peak amplitude ≥0.2 mV (Mahida et al., 2015; Tikkanen et al., 2009, 2011) and lower T/R ratio <0.2 (Roten et al., 2016). For MCG parameters, we used the Youden index to determine the cutoff values. We performed all analyses using JMP Pro^®^ 13 software (SAS Institute Inc.). All reported *p* values were two‐sided, and the significance level was set at *p* < .05.

## 3 RESULTS

### 3.1 MCG parameters of ERP‐VF(+) and ERP‐VF(−)

Among the source population of 2,636 consecutive subjects who had undergone MCG, 25 were survivors from VF without structural or electrical heart disorders, including 13 with ERP on ECG (ERP‐VF(+)). We also identified 103 ERP subjects without structural or electrical heart disorders (ERP‐VF(−)) (Figure S2).

Table 1 summarizes the clinical characteristics of total 116 study subjects (43 ± 18 years old, 54% male) without any missing data. Male gender was significantly more common in the ERP‐VF(+) group than in the ERP‐VF(−) group (12/13 [92%] vs. 51/103 [50%]; *p* = .003). The QTc interval was significantly shorter in the ERP‐VF(+) group than in the ERP‐VF(−) group (395 ± 19 vs. 414 ± 23 ms, *p* = .01). However, in male subjects only, the QTc interval did not differ between the two groups (393 ± 17 vs. 402 ± 21 ms, *p* = .15). No further difference could be observed between the two groups regarding existing ECG parameters such as J‐wave distribution (type 1 or type 2), J‐wave configuration (notch or slur), J‐peak amplitude, ST morphology, and T/R ratio. Only one (8%) of the 13 subjects with ERP‐VF(+) showed positive late potential of SAECG. SAECG was not always performed in ERP‐VF(−) group; thus, group comparison could not be done. Detailed ECG findings of all ERP‐VF(+) subjects are shown in Table S1 and Figure S3.

**Table 1:** Clinical characteristics of ERP‐VF(+) and ‐VF(−) subjects

Variables	ERP‐VF(+) (*n* = 13)	ERP‐VF(−) (*n* = 103)	*p* value
Age, mean(*SD*), year	39 (15)	44 (19)	.39
Gender, male, *n* (%)	12 (92)	51 (50)	.003
Family history of SCD, *n* (%)	2 (15)	4 (4)	.13
History of syncope, *n* (%)	0 (0)	17 (17)	.21
ECG findings			
J‐wave distribution			
Type 1, *n* (%)	2 (15)	10 (10)	.62
Type 2, *n* (%)	11 (85)	93 (90)	
J‐wave configuration			
Notch, *n* (%)	6 (46)	41 (40)	.77
Slur, *n* (%)	7 (54)	62 (60)	
J‐peak amplitude, mean (*SD*), mV	0.19 (0.09)	0.19 (0.10)	.18
ST morphology			
Descending or horizontal, *n* (%)	1 (8)	7 (7)	1.00
Ascending, *n* (%)	12 (92)	96 (93)	
T/R‐wave ratio in lead II (*SD*)	0.31 (0.17)	0.26 (0.13)	.20
T/R‐wave ratio in lead V_5_ (*SD*)	0.34 (0.15)	0.28 (0.16)	.17
Lower T/R‐wave ratio (lead II or V_5_) (*SD*)	0.27 (0.09)	0.23 (0.12)	.23
QRS duration, mean(*SD*), ms	102 (7)	99 (10)	.19
QTc interval, mean(*SD*), ms	395 (19)	414 (23)	.01
MCG findings			
MCG‐QRS, mean(*SD*), ms	108 (24)	91 (23)	.02
MCG‐RMS, mean(*SD*)	0.10 (0.08)	0.25 (0.20)	.01
MCG‐LAS, mean(*SD*), ms	38 (22)	29 (23)	.17

Note

Continuous variables are presented as means (*SD*) if normally distributed and as medians (interquartile range) if not normally distributed. Categorical variables are presented as numbers of patients (%).

Abbreviations: ECG, electrocardiography; ERP, early repolarization pattern; LAS, low amplitude signal; MCG, magnetocardiography; QTc, corrected QT; RMS, root‐mean‐square; SCD, sudden cardiac death; VF, ventricular fibrillation.

**Figure 2:**
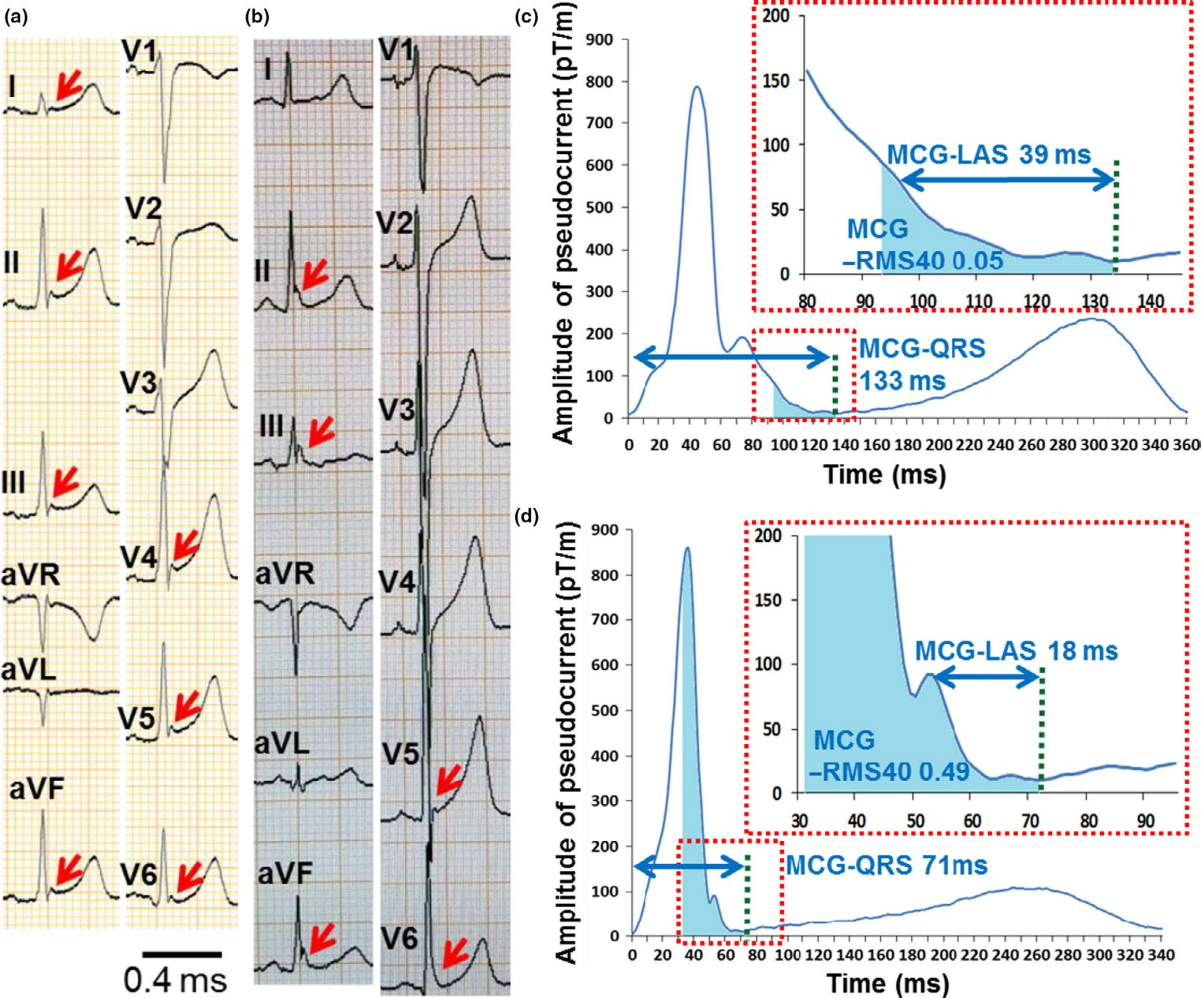
Cases of notched ERP with and without VF. (a, c) Thirty‐one‐year‐old male survivor of VF. (a) 12‐lead ECG shows notched ERP of 0.2 mV in inferolateral leads with ascending ST segment. (c) Terminal QRS complex in MCG waveform presents a characteristic gentle downslope. (b, d) Forty‐eight‐year‐old human without VF. (b) Twelve‐lead ECG shows notched ERP of 0.3 mV in inferolateral leads with ascending ST segment. (d) Terminal QRS complex in MCG waveform presents a steep downslope

**Figure 3:**
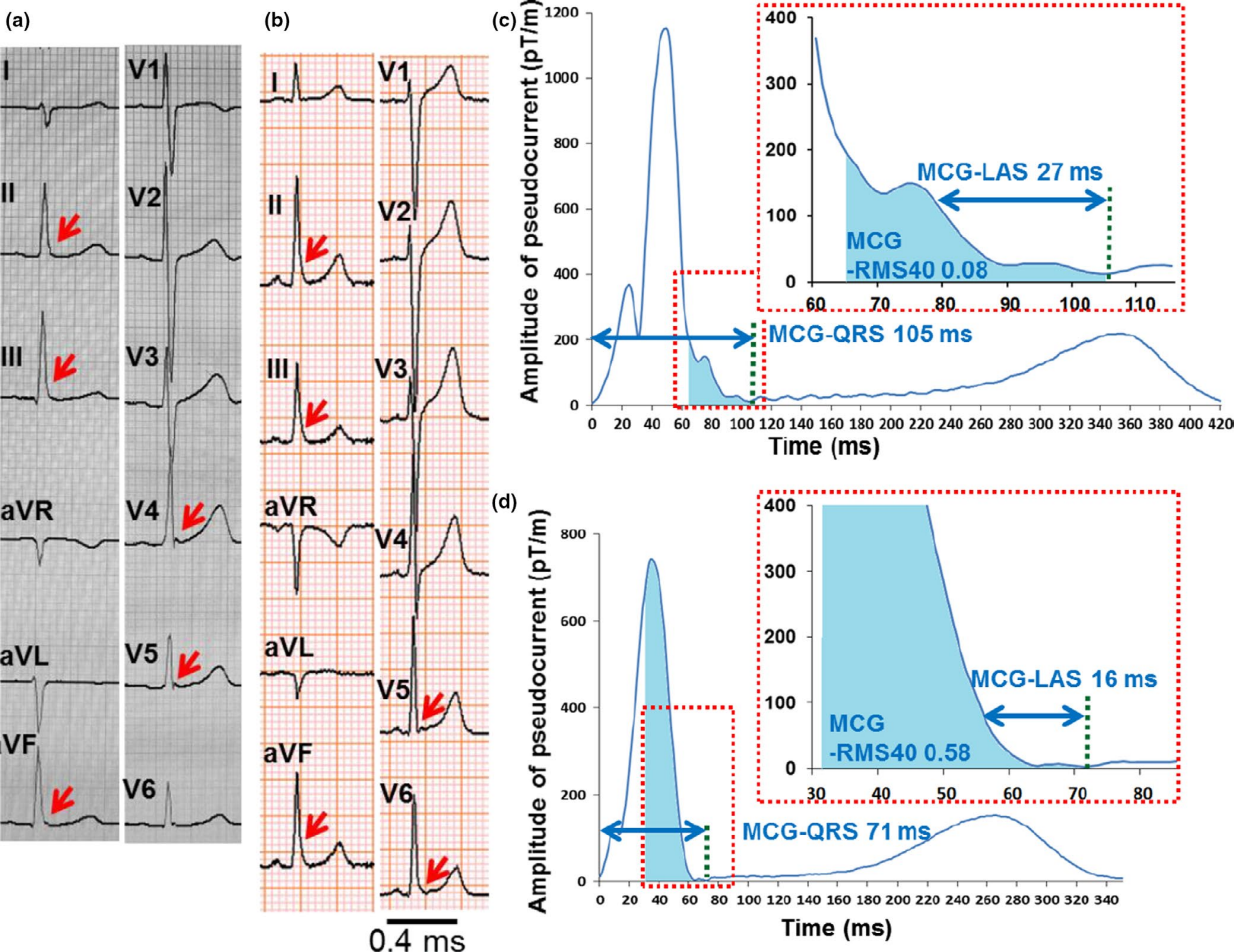
Cases of slur ERP with and without VF. (a, c) Eighteen‐year‐old male survivor of VF. (a) Twelve‐lead ECG shows slur ERP of 0.2 mV in inferolateral leads with ascending ST segment. (c) MCG waveform. (b, d) Forty‐six‐year‐old human without VF. (b) Twelve‐lead ECG shows slur ERP of 0.4 mV in inferolateral leads with ascending ST segment. (d) MCG waveform. ECG, electrocardiography; LAS, low signal amplitude; Max, maximal amplitude; MCG, magnetocardiography; Min, minimal amplitude; RMS, root‐mean‐square; SQUID, superconducting quantum interference device

Figures 2 and 3 show the 12‐lead ECGs and MCGs of representative cases. In Figure 2, both ERP‐VF(+) (Figure 2a,c) and ERP‐VF(−) subject (Figure 2b,d) had similar “notched”‐type ERPs in ECG, but MCG‐QRSD and MCG‐LAS were longer and MCG‐RMS40 was smaller in the ERP‐VF(+) subject (MCG‐QRSD: 133 vs. 71 ms, MCG‐LAS: 39 vs. 18 ms, and MCG‐RMS40: 0.05 vs. 0.49, respectively). Similarly, in Figure 3, both ERP‐VF(+) (Figure 3a,c) and ERP‐VF(−) subject (Figure 3b,d) had “slur”‐type ERPs and risk identification was difficult in ECG, but the MCG showed distinct difference (MCG‐QRSD: 105 vs. 71 ms, MCG‐LAS: 27 vs. 16 ms, and MCG‐RMS40: 0.08 vs. 0.58, respectively).

Overall, MCG‐QRSD was significantly larger (108 ± 24 vs. 91 ± 23 ms, *p* = .02) and MCG‐RMS40 was significantly smaller (0.10 ± 0.08 vs. 0.25 ± 0.20, *p* = .01) in the ERP‐VF(+) group compared to the ERP‐VF(−) group, but no difference in MCG‐LAS was seen between the two groups (38 ± 22 vs. 29 ± 23 ms, *p* = .17; Table 1). Figure S4 shows the location of the maximal amplitude channel in each subject. In most subjects (113 of 116; 97%), these channels were located in the previously determined LV area.

### 3.2 MCG parameters in subjects without ERP

Since all the VF survivors received MCG only after they survived the VF events, the impact of events on MCG parameters needs to be investigated. For this purpose, we additionally analyzed subjects with no ERP on ECG including 12 VF survivors (ERP(−)‐VF(+)) and 342 non‐VF subjects (ERP(−)‐VF(−)). As shown in Table S2, no significant difference was observed in the MCG parameters between the ERP(−)‐VF(+) and ERP(−)‐VF(−), suggesting that the MCG differences between ERP‐VF(+) and ERP‐VF(−) were not due to the VF event itself but exclusively to the arrhythmic substrate of ERP.

### 3.3 Comparison of ECG and MCG parameters

Table 2 shows the results of univariable and multivariable logistic analyses comparing ECG and MCG parameters. None of the 12‐lead ECG parameters were associated with the occurrence of VF. In contrast, MCG‐QRSD and MCG‐RMS40 were significantly associated with VF in univariable analysis, though MCG‐LAS was not. The three MCG parameters (MCG‐QRSD, MCG‐RMS40, and MCG‐LAS) were conceptually and statistically interrelated (*r* = .76, *p* < .001 for MCG‐QRSD and MCG‐RMS40, *r* = .89, *p* < .001 for MCG‐QRSD and MCG‐LAS) and were represented by MCG‐QRSD in the multivariable analysis. Consequently, MCG‐QRSD was the only parameter that was significantly associated with VF events.

**Table 2:** Logistic analysis comparing ECG and MCG parameters associated with VF

Parameters	Univariable OR (95% CI)	*p* value	Multivariable OR (95% CI)	*p* value
J‐wave distribution: type 2 versus type 1	0.59 (0.11–3.05)	.55	0.47 (0.07–3.07)	.35
J‐wave configuration: *notch* versus *slur*	1.30 (0.41–4.13)	.66	0.96 (0.25–3.64)	.95
J‐peak amplitude, mV	0.66 (0.001–323)	.89	1.34 (8.14 × 10^–4^–2,194)	.94
ST morphology: descending or horizontal versus ascending	1.14 (0.13–10.1)	.91	1.75 (0.18–17.4)	.65
Lower T/R‐wave ratio (lead II or V_5_)	15.3 (0.17–1,383)	.25	25.4 (0.17–3,901)	.22
MCG‐QRS, ms	1.02 (1.003–1.05)	.02	1.03 (1.004–1.05)	.02
MCG‐RMS	3.24 × 10^–3^ (2.60 × 10^–5^–0.40)	.004	–	–
MCG‐LAS, ms	1.02 (0.99–1.04)	.20	–	–

Abbreviations: CI, confidence interval; ECG, electrocardiography; LAS, low amplitude signal; MCG, magnetocardiography; OR, odds ratio; RMS, root‐mean‐square; VF, ventricular fibrillation.

**Figure 4:**
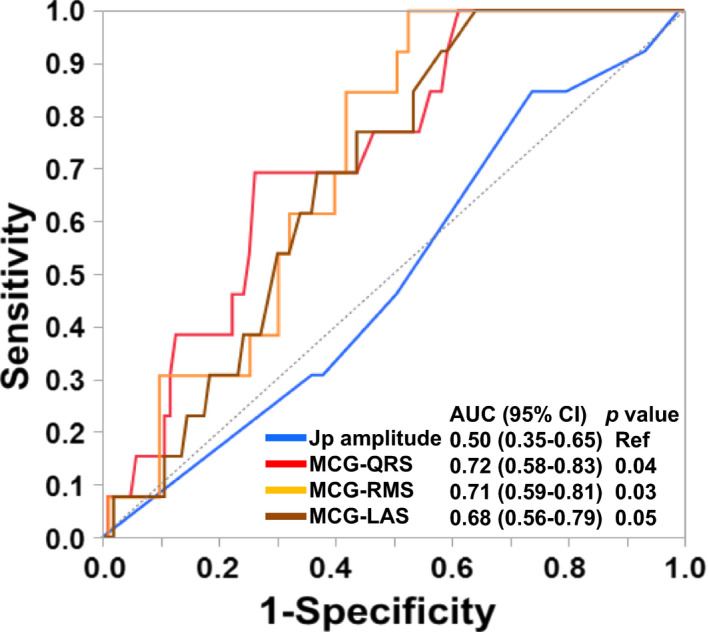
Receiver operating characteristic analysis of ECG and MCG parameters. MCG‐QRSD and MCG‐RMS40 showed significantly larger AUC than did J‐peak amplitude in ECG. AUC, area under curve; Jp, J peak; LAS, low signal amplitude; MCG, magnetocardiography; Ref, reference; RMS, root‐mean‐square

We further compared the performance of the parameters by area under ROC curves. Compared to the J‐peak amplitude, MCG‐QRSD (AUC = 0.72, *p* = .04) and MCG‐RMS40 (AUC = 0.71, *p* = .03) showed significantly larger AUC, though MCG‐LAS (AUC = 0.68, *p* = .05) did not (Figure 4). The cutoff criteria determined by means of the Youden index were MCG‐QRSD ≥ 100 ms and MCG‐RMS40 ≤ 0.24, which yielded sensitivity, specificity, and odds ratios of 69 and 74%, 92 and 48%, and 6.33 (95% CI = 1.80–22.3, *p* = .003) and 10.9 (95% CI = 1.37–86.8, *p* = .007), respectively (Table 3).

**Table 3:** Performance of ECG and MCG parameters to detect malignant ERPs

Parameters and cutoffs	Sensitivity, %	Specificity, %	Positive likelihood ratio	Negative likelihood ratio	Odds ratio (95% CI)	*p* value
ECG parameters						
J‐wave distribution: type 2	85	10	0.94	1.58	0.59 (0.11–3.05)	.62
J‐wave configuration: notch	46	60	1.16	0.89	1.30 (0.41–4.13)	.77
J‐peak amplitude ≥0.2 mV	54	50	1.09	0.91	1.19 (0.38–3.78)	1.00
ST morphology: descending or horizontal	8	93	1.13	0.99	1.14 (0.13–10.1)	1.00
Lower T/R‐wave ratio (lead II or V_5_) <0.2	15	49	0.30	1.74	0.17 (0.04–0.81)	.02
MCG parameters						
MCG‐QRS ≥ 100 ms	69	74	2.64	0.42	6.33 (1.80–22.3)	.003
MCG‐RMS ≤ 0.24	92	48	1.76	0.16	10.9 (1.37–86.8)	.007
MCG‐LAS ≥ 18 ms	100	36	1.56	0	–	–

Abbreviations: CI, confidence interval; ECG, electrocardiography; ERP, early repolarization pattern; LAS, low amplitude signal; MCG, magnetocardiography; OR, odds ratio; RMS, root‐mean‐square; VF, ventricular fibrillation.

## 4 DISCUSSION

### 4.1 Major findings

The present case–control study demonstrated that MCG‐QRSD and MCG‐RMS40, a characteristic waveform of the terminal QRS complex in the maximal amplitude channel of MCG, but none of the existing 12‐lead ECG parameters, can be used to distinguish malignant from benign ERP. To the best of our knowledge, this is the first study to show MCG‐based noninvasive risk stratification for VF in subjects with ERP.

### 4.2 Difference between ECG and MCG

Magnetocardiography is a noncontact and noninvasive instrument for recording the magnetic fields generated by cardiac electrical activity. Both ECG and MCG observe the same activity; however, MCG has exclusive advantages for detecting abnormal cardiac currents. According to the Helmholtz's theorem, any vector field in three dimensions can be resolved into the sum of a divergence and curl vector field. MCG is superior in detecting latter current vector. An ideal closed loop current does not produce electric field reaching the body surface, but is apparent in MCG (Liehr et al., 2005). ECG is more sensitive to electrical currents radial to the body surface, while MCG is more sensitive to currents tangential to it (Barry et al., 1977; Nousiainen et al., 1986). Tangential currents contribute more at the terminal phase of normal depolarization and also around the pathological area. With these unique features, MCG has shown superior performance in detecting abnormal currents when compared with standard 12‐lead ECG or SAECG.

### 4.3 ERP: malignant or benign?

The pathophysiological role of ERP is still unclear. Some ECG characteristics such as distribution, configuration, and peak amplitude of ERPs (Tikkanen et al., 2011); T/R ratios (Roten et al., 2016); and morphology of the ST segments (Rosso et al., 2012) have been reported as potential tools to stratify the risk of VF. However, their predictive values are insufficient to enable clinicians to identify malignant ERPs from among the numerous benign ERPs (Arbelo & Brugada, 2015; Mahida et al., 2015). In this study, even though no significant difference between the ERP‐VF(+) and ERP‐VF(−) groups was observed in any of the 12‐lead ECG parameters, significant differences were observed in MCG parameters: MCG‐QRSD was significantly larger and MCG‐RMS40 was significantly smaller in the ERP‐VF(+) group compared with the ERP‐VF(−) group.

Early repolarization pattern is frequently observed in patients with short QT syndrome or Brugada syndrome and is potentially a marker for increased risk of VF and sudden cardiac death in these disease entities (Kamakura et al., 2009; Watanabe et al., 2010). Furthermore, in inferolateral early repolarization syndrome, coexistence of non‐type 1 anterior ERP is associated with VF (Kamakura et al., 2013). In this study, although the QTc interval was shorter in the ERP‐VF(+) group than in the ERP‐VF(−) group, this difference may reflect the predominance of male subjects in the ERP‐VF(+) group. Furthermore, as there was no Brugada pattern in the anterior leads of ECG (Figure S3) in ERP‐VF(+) subjects, this study completely excluded short QT and Brugada syndromes and other arrhythmic syndromes.

### 4.4 Comparison of SAECG and MCG parameters

Signal‐averaged electrocardiography can unmask abnormal delayed ventricular activation at the end of the QRS, not only in some structural heart diseases but also in Brugada syndrome, and a positive late potential detected through SAECG is one of the useful markers for VF risk stratification (Adler et al., 2016; Huang et al., 2009). In contrast, the utility of SAECG is less established in patients with early repolarization syndrome or idiopathic VF. In a study of 687 subjects, Soliman, Elsalam, and Li (2012) demonstrated no significant difference in SAECG parameters between subjects with and subjects without ERP. In that study, the SAECG parameters reported for the ERP subjects (comparable to the ERP‐VF(−) subjects in the present study) were as follows: filtered QRS duration (fQRS): 106 ± 10 ms, RMS40: 27 ± 16 µV, and LAS40: 34 ± 9 ms, with 11% of subjects showing positive late potentials. These data are similar to the results seen in the ERP‐VF(+) subjects in the present study, which leads us to conclude that SAECG has a limited ability to predict sudden cardiac death in subjects with ERP.

### 4.5 Mechanisms of ERP: electrical abnormalities in the left ventricle?

The electrophysiological mechanism of ERP is still controversial, involving either depolarization or repolarization (Antzelevitch & Yan, 2010; Borggrefe & Schimpf, 2010). There is no established MCG method to clearly discriminate depolarization and repolarization signals; however, late field potentials detected in this study had continuity with depolarization signals. Idiopathic VF patients with ERP had a high incidence of late potentials showing a circadian variation with night ascendancy though repolarization abnormalities such as T‐wave alternans and QT dispersion were not observed, suggesting that ERP may be more closely associated with depolarization abnormality and autonomic modulation than with repolarization abnormality (Abe et al., 2010). On the other hand, Koncz et al. (2014) demonstrated using coronary‐perfused canine LV wedge preparations that ERP could be caused by a preferential accentuation of the action potential notch in the LV. Benito, Guasch, Rivard, and Nattel (2010) also explained that regional or transmural difference in action potentials in LV was the underlying mechanism of ERP and its VF risk. Boineau (2007) noted that deep penetration of the Purkinje network into the LV wall induced regional shortening of transmural activation time with regional electrical heterogeneity. Haissaguerre et al., (2008) reported that most of their ERP‐VF(+) cases presented LV ectopies which could have triggered VF. These findings suggest that some structural and/or electrical substrates of ERP in the LV may underlie VF.

Most of the channels of maximal amplitude in MCG fell exactly in the LV area (Figure S4), and risk detection was possible when focusing on signals in the LV. Therefore, we assume that the late field potentials that we measured using MCG, namely MCG‐QRSD and MCG‐RMS40, are associated with regional electrical activity in the LV.

### 4.6 Study limitations

We had some limitations in this retrospective case–control study. First, the sample size was so small that multivariable analysis might suffer from overfitting. Second, the source population is a hospital cohort and may be biased to some extent. The control subjects (ERP(+)‐VF(−)) were not representative of the general population with ERP because they were recruited in a hospital; although no electrical and structural heart diseases were found in this population, they had some reasons to visit the hospital, such as history of syncope or family history of sudden cardiac death. None of the control subjects had experienced a VF event at the time of MCG, but follow‐up data, which were not available in this study, might have revealed subsequent VF events. This selection bias might lead us to underestimate the values of the ECG and MCG parameters. Therefore, a prospective population‐based cohort study would be necessary to conclude whether our MCG parameters are useful to predict future VF events in general ERP subjects.

## 5 CONCLUSIONS

Magnetocardiography‐based parameters including QRS and RMS, but not the standard 12‐lead ECG parameters, are useful as screening tools to distinguish malignant from benign ERP, suggesting that MCG is useful in risk stratification of sudden cardiac death in subjects with ERP.

## CONFLICTS OF INTEREST

None.

## AUTHOR CONTRIBUTIONS

Conceived and designed the analysis: NI, TA, SK. Collected the data: NI, TA, SK, HT, YI, SN, WS, SY, MS, KK. Contributed data or analysis tools: HT, WS, TS. Performed the analysis: TAF, TS, KN. Wrote this paper: NI, TA. All authors reiviewed and approved the manuscript.

## ETHICS

All procedures performed in this study were in accordance with the ethical standards of the institutional and national research commitee and with the 1964 Helsinki declaration and its later amendments. The institutional review board at the National Cerebral and Cardiovascular Center approved this MCG study with waivers of individual consent (M23‐050, M24‐050‐6). The ethics committee at Kyoto University, where the analysis was conducted, approved the use of the cohort data for this analysis (E2321).
