# Supplementary material for: Identification of malignant early repolarization pattern by late QRS activity in high‐resolution magnetocardiography
Source: Ann Noninvasive Electrocardiol. 2020 Jan 19;25(4):e12741. doi: 10.1111/anec.12741 (PMC7358799; doi:10.1111/anec.12741)
Supplement: Supplementary file 1 [file ANEC-25-e12741-s001.docx]

**Supplemental Materials**

**Supplemental Methods.**

**Supplemental Figure S1.** MCG study and definition of MCG parameters

**Supplemental Figure S2.** Flow chart of the subject enrollment

**Supplemental Figure S3.** ECGs of ERP-VF(+) subjects

**Supplemental Figure S4.** Location of a MCG channel with maximal amplitude

**Supplemental Table S1.** Clinical characteristics of ERP-VF(+) subjects

**Supplemental Table S2.** Clinical characteristics of ERP(-)-VF(+) and ERP(-)-VF(-) subjects

**Supplemental Methods**

**12-lead ECG and SAECG**

A standard 12-lead ECG (Cardiofax V ECG-9321, Nihon Kohden Corp., Tokyo, Japan or FCP-7541, Fukuda Denshi, Tokyo, Japan) was conducted within a week of MCG study with a setting of 0.05–150-Hz filter, 60-Hz AC notch filter, 25 mm/s, and 10 mm/mV. We obtained the following ECG data: distribution, configuration and peak amplitude of the ERP, ST morphology, T-wave / R-wave (T/R) ratio, and QRS and QTc interval as defined in a 2015 consensus paper.([1](#_ENREF_1)) ERP distribution was classified as described in a previous paper ([2](#_ENREF_2)), with type 1 consisting of lateral ERPs, type 2 of infero-lateral ERPs, and type 3 of infero-lateral and right precordial ERPs, though type 3 was not included in this study. The T/R-amplitude ratio was calculated in lead II and V5 ([3](#_ENREF_3)) and the QTc interval was calculated by heart rate with Bazett’s formula.([4](#_ENREF_4)).

SAECG was recorded using a commercially available system (1200 EXP, Arrhythmia Research Technology, Fitchburg, MA, USA). We averaged over 350–400 beats using a bidirectional 40-Hz filter. The noise level was < 0.3 μV. The following parameters were measured: filtered QRS duration (fQRS), root-mean-square voltage in the terminal 40 ms (RMS40), and duration of the low amplitude signal <40 μV (LAS40). Late potential was defined as present when more than two of the following criteria were met: fQRS >114 ms, RMS40 <20 μV, and LAS40 >38 ms.

**MCG recording**

We used a 64-channel MCG system (MC-6400, Hitachi High-Technologies Ltd., Tokyo, Japan) with highly sensitive sensors of superconducting quantum interference device (SQUID) arranged in an 8*8 matrix with 25-mm pitch and 175*175 mm measurement area (**Supplemental Figure 1A**). MCG signals for each subject were recorded in the resting supine position from the frontal planes in a magnetically shielded room for more than 30 seconds (**Supplemental Figure 1B**). The detected signals were passed through an analog bandpass filter (0.1–100 Hz) and an analog notch filter (60 Hz). They were subsequently digitized at a sampling rate of 1 kHz by an analog-digital converter. To remove the noise in the signals, the MCG data were signal-averaged 30 times using a trigger of simultaneously recorded ECG signals. The tangential component of measured magnetic fields (Bz) were then transformed into pseudo electrical currents (C) to show the current arrow map by the Hosaka-Cohen transformation([5](#_ENREF_5)):

$$\vec{C}=\frac{\partial B_{z}}{\partial y}\vec{e}_{x}-\frac{\partial B_{z}}{\partial x}\vec{e}_{y}.$$

The unit vectors x- and y-direction are described as e_x_ and e_y_. Time-domain waveforms of their magnitudes were drawn for each channel (**Supplemental Figure 1C**).

We focused on the waveform with maximal amplitude. We defined the end of the QRS complex as the minimal amplitude point, and the onset was automatically determined by the simultaneously recorded ECG (**Supplemental Figure 1D**). We then measured the following three parameters: (1) MCG-QRSD; the interval between the onset and end of QRS complex, (2) MCG-RMS40; the root mean square (RMS) amplitude of the terminal 40ms divided and corrected by the maximal amplitude, and (3) MCG-LAS; the duration of low amplitude signal (LAS) at the terminal portion of the QRS complex under 10% of maximal amplitude. All parameters were automatically calculated from raw data by a computing algorithm. We additionally investigated the location of the maximal amplitude channel referring to the previously reported regional map of MCG.([6](#_ENREF_6)) For VF survivors, MCG was performed within weeks after the VF events, as soon as the patients were stabilized without drugs or assistive devices. For subjects without VF, the timing of the examination was at the physician’s discretion in accordance with the patient’s availability and convenience.

**Online References**

1. Macfarlane PW, Antzelevitch C, Haissaguerre M, Huikuri HV, Potse M, Rosso R, et al. The Early Repolarization Pattern: A Consensus Paper. J Am Coll Cardiol. 2015;66(4):470-7.

2. Antzelevitch C, Yan GX**.** J wave syndromes. Heart Rhythm. 2010;7(4):549-58.

3. Roten L, Derval N, Maury P, Mahida S, Pascale P, Leenhardt A, et al. Benign vs. malignant inferolateral early repolarization: Focus on the T wave. Heart Rhythm. 2016;13(4):894-902.

4. Bazett H**.** An analysis of the time-relations of electrocardiograms. Heart. . 1920;7:353-70.

5. Hosaka H, Cohen D**.** Part IV: visual determination of generators of the magnetocardiogram. J Electrocardiol. 1976;9(4):426-32.

6. Kandori A, Ogata K, Miyashita T, Watanabe Y, Tanaka K, Murakami M, et al. Standard template of adult magnetocardiogram. Ann Noninvasive Electrocardiol. 2008;13(4):391-400.

**Supplemental Figures**

**Supplemental Figure S1. MCG study and definition of MCG parameters**

**
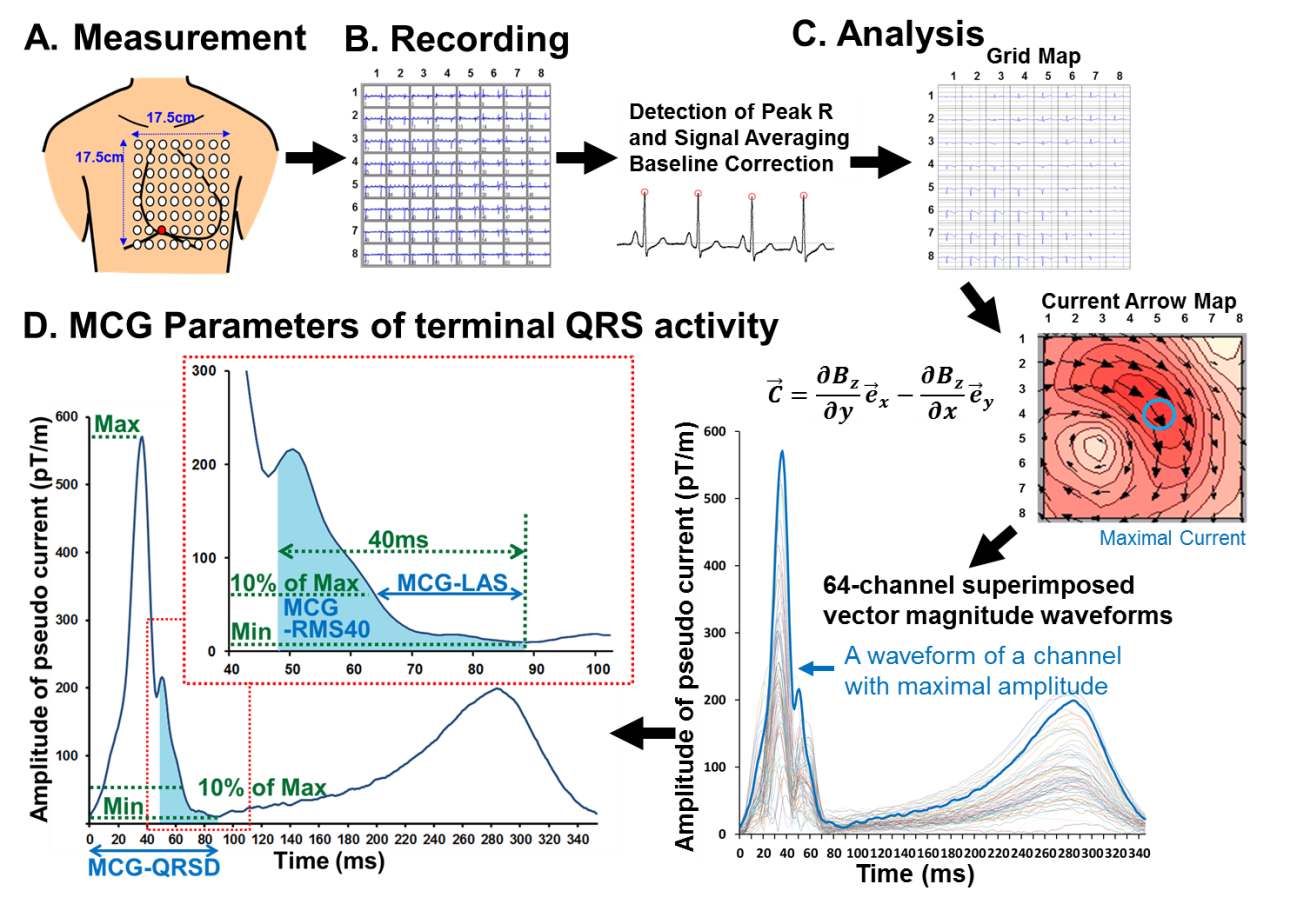
**

**A,B**: 64 (8*8) SQUID sensors arranged with 25 mm pitch were placed close to each subject’s chest in a supine position, with a marking sensor (red circle) on the xiphisternum.

**C**: After signal filtering and baseline correction, the measured magnetic fields (Bz) were mathematically transformed into psudo electrical currents (C) following Hosaka-Cohen transformation. The unit vectors x- and y-direction are described as e_x_ and e_y_. Time-domain waveforms of current magnitudes in 64 channels were superimposed and the maximal peak amplitude channel was used for analysis.

**D**: Definition of the three parameters at the end of the QRS complex. QRS end was defined as the minimal amplitude point. (1) MCG-QRSD; the QRS duration. (2) MCG-RMS40; the root-mean-square amplitude of the terminal 40ms corrected by the maximal amplitude. (3) MCG-LAS; the duration of low signal amplitude at the terminal QRS under 10% of maximal amplitude.

ECG: electrocardiography; LAS: low signal amplitude; MCG: magnetocardiography; RMS: root mean square; SQUID: superconducting quantum interference device

**Supplemental Figure S2. Flow chart of the subject enrollment**

**
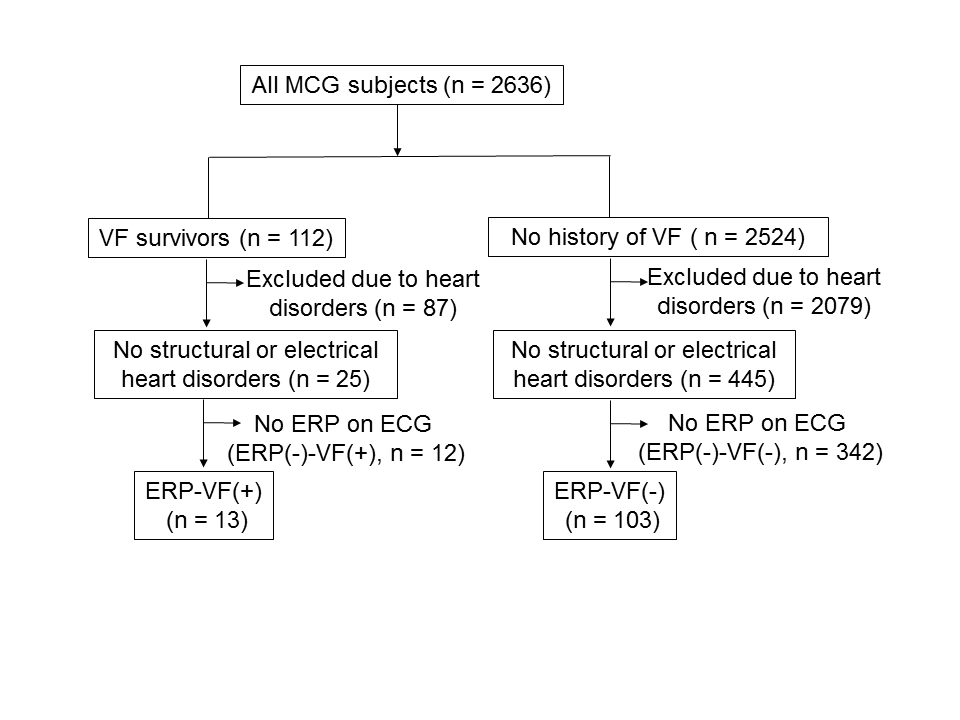
**

ECG = electrocardiography; ERP = early repolarization pattern; MCG = Magnetocardiography; VF = ventricular fibrillation.

**Supplemental Figure S3. ECGs of all ERP-VF(+) subjects**


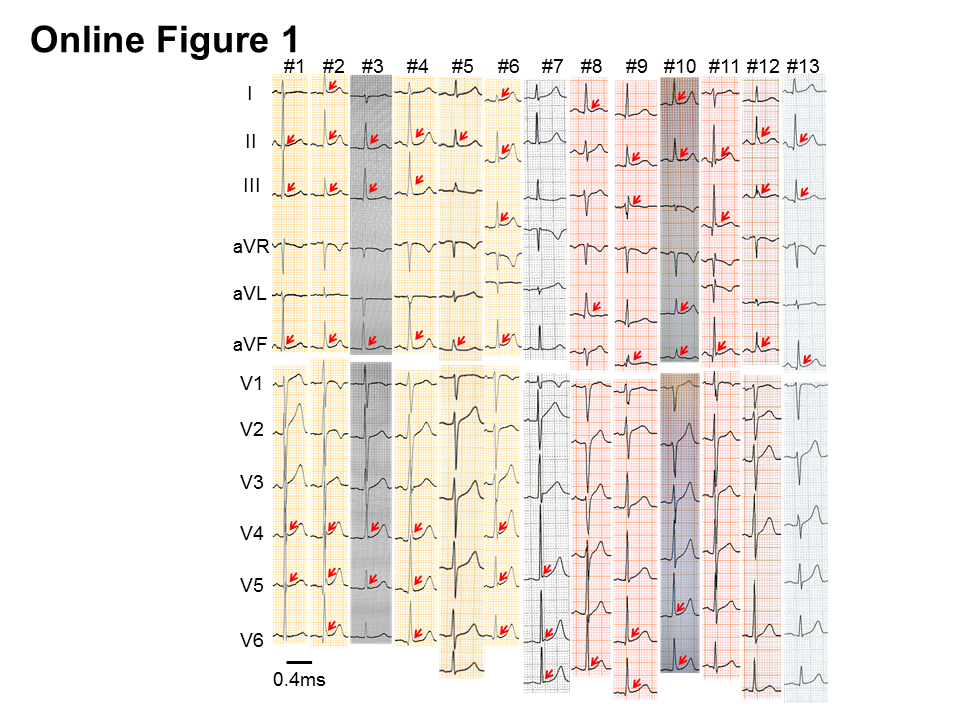


Red arrows indicate early repolarization patterns.

ECG = electrocardiography; ERP = early repolarization pattern; VF = ventricular fibrillation.

**Supplemental Figure S4. Location of a MCG channel with maximal amplitude**


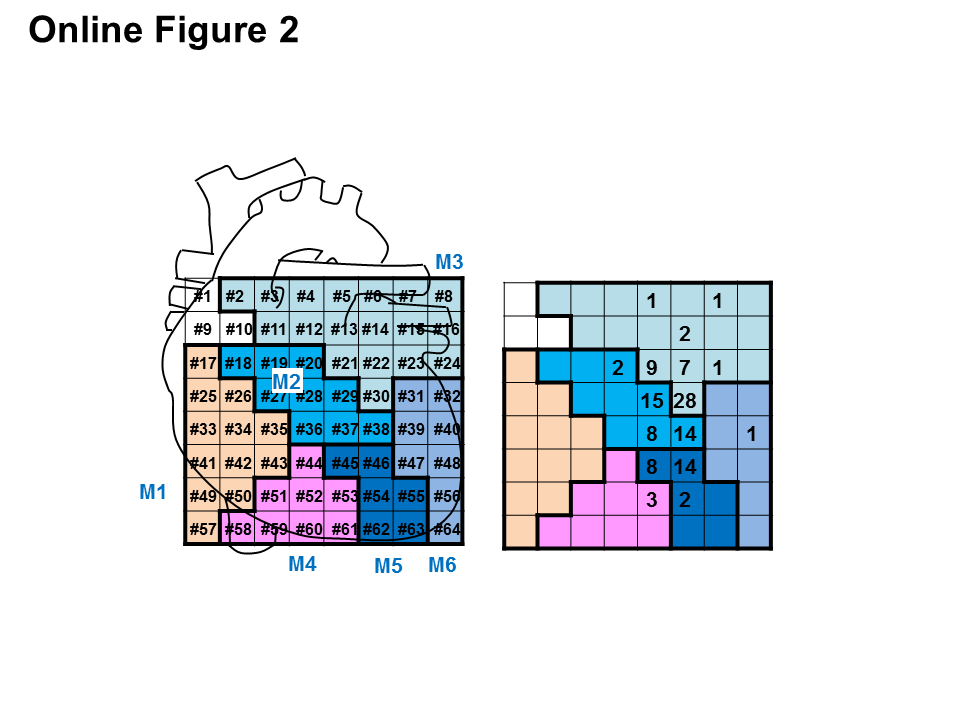


**Left**: MCG 64 channels were numbered. Each colored area corresponds to area of the heart shown by Kandori et al.([6](#_ENREF_6)) M1 indicates right atrium, M2 indicates septum and mid left ventricle, M3 indicates basal left ventricle, M4 indicates right ventricle, M5 indicates apex and lower-left ventricle, and M6 indicates lateral wall of left ventricle.

**Right**: A MCG channel with maximal amplitude was plotted and counted in 116 study subjects.

MCG = magnetocardiography.

**Supplemental Table S1. Clinical characteristics of all ERP-VF(+) subjects**

| # | Age^*^ | Sex | J-wave distribution | Notch/ slur | J peak, mV | ST morphology | QTc,  ms | fQRS, ms | RMS40, µV | LAS40, ms | SAECG  LP | MCG-  QRSD, ms | MCG-RMS40 | MCG-  LAS, ms |
| --- | --- | --- | --- | --- | --- | --- | --- | --- | --- | --- | --- | --- | --- | --- |
| 1 | 39 | M | Inferolateral | notch | 0.10 | ascending | 394 | 89 | 17 | 30 | negative | 81 | 0.22 | 31 |
| 2 | 63 | M | Inferolateral | slur | 0.30 | ascending | 394 | 105 | 53 | 32 | negative | 102 | 0.07 | 34 |
| 3 | 18 | M | Inferolateral | slur | 0.20 | ascending | 403 | 87 | 28 | 7 | negative | 105 | 0.08 | 27 |
| 4 | 34 | M | Inferolateral | slur | 0.40 | ascending | 404 | 101 | 43 | 32 | negative | 115 | 0.02 | 44 |
| 5 | 44 | M | Inferior | notch | 0.10 | horizontal | 385 | 104 | 31 | 33 | negative | 122 | 0.02 | 51 |
| 6 | 31 | M | Inferolateral | notch | 0.20 | ascending | 381 | 105 | 37 | 25 | negative | 133 | 0.05 | 39 |
| 7 | 34 | M | Inferior | notch | 0.15 | ascending | 381 | 104 | 27 | 26 | negative | 89 | 0.15 | 20 |
| 8 | 38 | M | Lateral | slur | 0.10 | ascending | 362 | 118 | 15 | 50 | positive | 167 | 0.02 | 98 |
| 9 | 37 | M | Inferolateral | slur | 0.20 | ascending | 380 | 104 | 27 | 26 | negative | 100 | 0.22 | 31 |
| 10 | 49 | M | Inferolateral | slur | 0.20 | ascending | 402 | 104 | 19 | 42 | negative | 117 | 0.01 | 59 |
| 11 | 17 | M | Inferior | notch | 0.15 | ascending | 429 | 105 | 18 | 37 | negative | 85 | 0.17 | 18 |
| 12 | 68 | M | Inferior | slur | 0.20 | ascending | 398 | 94 | 39 | 26 | negative | 82 | 0.24 | 21 |
| 13 | 38 | F | Inferior | notch | 0.10 | ascending | 429 | 125 | 29.8 | 32 | negative | 100 | 0.09 | 34 |

* Age when the MCG was performed.

ERP = early repolarization pattern, F = female, fQRS = filtered QRS duration; LAS = low amplitude signal; LP = late potential; M = male; MCG = magnetocardiography; QRSd = filtered QRS duration; QTc = corrected QT; RMS = root mean square; SAECG = signal averaged electrocardiography; VF = ventricular fibrillation; # = subject number.

**Supplemental Table S2. Clinical characteristics of ERP(-)-VF(+) and ERP(-)-VF(-) subjects**

| Variables | **ERP(-)-VF(+)**  (n = 12) | **ERP(-)-VF(-)**  (n = 342) | *P* value |
| --- | --- | --- | --- |
| Age, mean (SD), year | 44 (14) | 45 (18) | 0.89 |
| Gender, male, n (%) | 10 (83) | 160 (45) | 0.02 |
| Family history of SCD, n (%) | 0 (0) | 5 (1) | 1.00 |
| History of syncope, n (%) | 1 (8) | 12 (4) | 0.37 |
| **ECG findings** |  |  |  |
| QRS duration, mean (SD), ms | 94 (8) | 95 (10) | 0.70 |
| QTc interval, mean (SD), ms | 415 (23) | 417 (21) | 0.76 |
| **MCG findings** |  |  |  |
| MCG-QRSD, mean (SD), ms | 95 (26) | 92 (20) | 0.61 |
| MCG-RMS40, mean (SD) | 0.33 (0.23) | 0.23 (0.18) | 0.06 |
| MCG-LAS, mean (SD), ms | 29 (29) | 26 (17) | 0.60 |

Continuous variables are presented as means (SD) if normally distributed and as medians (interquartile range) if not normally distributed. Categorical variables are presented as numbers of patients (%).

ECG = electrocardiography; ERP = early repolarization pattern; LAS = low amplitude signal; MCG = magnetocardiography; QTc = corrected QT; RMS = root mean square; SCD = sudden cardiac death; VF = ventricular fibrillation.
